# A phase II clinical trial of 6-mercaptopurine (6MP) and methotrexate in patients with BRCA defective tumours: a study protocol

**DOI:** 10.1186/1471-2407-14-983

**Published:** 2014-12-19

**Authors:** Shibani Nicum, Corran Roberts, Lucy Boyle, Sylwia Kopijasz, Charlie Gourley, Marcia Hall, Ana Montes, Christopher Poole, Linda Collins, Anna Schuh, Susan J Dutton, on behalf of the 6MP Collaborative Group

**Affiliations:** Oxford University Hospitals NHS Trust, Oxford, UK; Centre for Statistics in Medicine, University of Oxford, Oxford, UK; OCTO – Oncology Clinical Trials Office, Department of Oncology, University of Oxford, Oxford, UK; Edinburgh Cancer Research Centre, Edinburgh, UK; Mount Vernon Cancer Centre, East and North Hertfordshire NHS Trust, Northwood, Middlesex, UK; Guy’s and St Thomas’ NHS Foundation Trust, London, UK; University Hospital Coventry & Warwickshire NHS Trust, Coventry, UK; Oxford Clinical Trials Research Unit (OCTRU), University of Oxford, Oxford, UK; Medical Oncologist, Department of Oncology, Churchill Hospital, Oxford, OX3 7LJ UK

**Keywords:** Breast cancer, Ovarian cancer, BRCA genes, Response

## Abstract

**Background:**

BRCA1 and BRCA2 genes are critical in homologous recombination DNA repair and have been implicated in familial breast and ovarian cancer tumorigenesis. Tumour cells with these mutations demonstrate increased sensitivity to cisplatin and poly (ADP-ribose) polymerase (PARP) inhibitors. 6MP was identified in a screen for novel drugs and found to selectively kill BRCA-defective cells in a xenograft model as effectively as the PARP inhibitor AGO14699, even after these cells had acquired resistance to a PARP inhibitor or cisplatin. Exploiting the genetic basis of these tumours enables us to develop a more tailored approach to therapy for patients with BRCA mutated cancers.

**Methods:**

This multi-centre phase II single arm trial was designed to investigate the activity and safety of 6-mercaptopurine (6MP) 55 mg/m^2^ per day, and methotrexate 15 mg/m^2^ per week in patients with advanced breast or ovarian cancer, ECOG PS 0–2, progressing after ≥ one prior regimen and known to bear a BRCA1/2 germ line mutation. Accrual was planned in two stages, with treatment continuing until progression or unacceptable toxicity; in the first, if less than 3/30 evaluable patients respond at 8 weeks after commencing treatment, the trial will be stopped for futility; if not, then accrual would proceed to a second stage, in which if more than 9/65 evaluable patients are found to respond at 8 weeks, the treatment will be regarded as potentially effective and a phase III trial considered subject to satisfactory safety and tolerability. The primary outcome is objective response at 8 weeks, defined by RECISTS v1.1 as complete response, partial response or stable disease. Secondary outcomes include safety, progression- free and overall survival, and quality of life.

**Discussion:**

This study aims to investigate whether 6MP might be an effective treatment for BRCA deficient tumours even after the development of resistance to PARP inhibitors or platinum drugs. The study has surpassed the first stage analysis criteria of more than 3 out of 30 evaluable patients responding at 8 weeks, and is currently in the second stage of recruitment.

**Trial registration:**

NCT01432145http://www.ClinicalTrials.gov

## Background

Ovarian cancer is the fourth most common cause of cancer mortality in women; the majority (75%) of ovarian cancer patients will present with late stage disease (International Federation of Gynaecology and Obstetrics (FIGO) stage III/IV), and the overall prognosis remains poor with five year survival rates of 30- 40%. Current first line chemotherapy for ovarian cancer usually comprises of a combination of carboplatin and paclitaxel. Despite complete remissions in approximately 75% of patients, responses are generally short lived, with median progression-free survival (PFS) ranging from 16–21 months [1]. Despite initial response rates of 65-80% to first line chemotherapy, the majority of patients will relapse and ultimately develop resistance to further chemotherapy.

Breast cancer is the most common cancer in the UK; around 55,000 people are diagnosed each year. There are currently multiple options for the treatment of patients with advanced breast cancer, including hormone therapy, chemotherapy and Her-2-directed therapy, as well as possible radiotherapy and/or surgery for patients with symptomatic oligo-metastatic disease, thus most breast cancer patients with advanced stage disease will receive multiple lines of therapy.

BRCA1 and BRCA2 genes play an important role in homologous recombination DNA repair and have been implicated in familial breast and ovarian cancer syndromes. In addition to germline BRCA1/2 mutations, silencing of BRCA1/2 expression can also occur via epigenetic processes such as promoter hyper- methylation and this has been documented in 11-14% of breast cancers [2] and 11-35% of ovarian cancer patients [3].

For patients with metastatic cancer, the challenge is to develop better treatment strategies to maximise tumour cell kill and minimise toxicity. In patients with BRCA1/2 deficient cancers, one such approach has been to develop molecular targeted therapy using PARP inhibitors that selectively exploit biological pathways within tumour cells, which differ from those in normal cells and wider concepts of genetic synthetic lethality [4–6]. PARP is a nuclear enzyme activated in response to DNA single strand breaks and is involved in repair of these lesions via the base excision pathway (BER). The main lesions formed by cisplatin are intra-strand crosslinks between closely adjacent purine bases [7]. Efficient BER is also required for repair of these lesions. An inability to couple DNA damage to an apoptotic signal pathway may lead to the development of resistance to platinum and PARP inhibitors. Tumours may also acquire resistance by additional mutations in the BRCA1 & BRCA2 gene [8–10] and so there is a place for further investigation of DNA repair inhibition in HR defective tumours.

Carriers of BRCA mutations have an increased lifetime risk of developing breast or ovarian cancer and in these families the focus has been on prevention (screening for early detection or prophylactic surgery). Hitherto, once cancers have developed in these patients they have generally received standard therapy for that tumour type. However patients with a BRCA1/2 mutation have an increased sensitivity to agents that cause DNA damage through double stranded breaks or DNA cross-links, such as platinum agents due to the genetic basis of their tumours. Recent clinical studies with cisplatin based chemotherapy and PARP inhibitors have sought to exploit the genetic basis of these tumours in order to develop a more tailored approach to therapy for patients with breast and ovarian cancer and known BRCA mutations and/or suspected defects in homologous recombination (defined as patients with triple receptor negative (oestrogen, progesterone and HER2 receptor-negative) breast cancer, or advanced high grade serous ovarian cancer with a history of repeated platinum sensitivity).

The thiopurines 6-mercaptopurine (6MP) and 6-thioguanine (6TG) are growth inhibitory antimetabolites licensed for the treatment of acute leukaemias and also chronic granulocytic leukaemia. Structurally, these agents are purine analogues that interfere with nucleic acid biosynthesis. Methotrexate is also an antimetabolite and inhibits tetrahydrofolate dehydrogenase and prevents formation of tetrahydrofolate, which is required for synthesis of thymidylate, an essential component of DNA. It is routinely used in combination with 6MP in patients with ALL as it has been shown to reduce de novo purine synthesis and thus enhance the cytotoxicity of 6MP by promoting its conversion to 6TGNs [11, 12]. Methotrexate has also been widely used in the treatment of a number of solid tumours such as breast, ovarian, lung and cervical cancers. Furthermore a recent synthetic lethality screen has identified methotrexate as an agent that has activity in DNA mismatch repair (MMR) defective cancers, as evidenced by its selective toxicity in cells lacking functional mutS homolog 2 (MSH2) gene mutations [13].

The rationale for our study is based on in-vitro work by Issaeva et al. [14] who have shown that 6- thioguanine (6TG) selectively kills BRCA2-defective cells as effectively as the PARP inhibitor, AGO14699. Even after cells have acquired resistance to a PARP inhibitor or cisplatin, they retain sensitivity to 6TG. However as long term administration of 6TG is problematic due to dose related toxicities, such as veno- occlusive disease, this study will assess the activity of 6-mercaptopurine (6MP), which is converted to the same active moiety, 6-thioguanine nucleotide, prior to incorporation into DNA. Low dose methotrexate will be used in combination with 6MP in this study as it promotes the formation of thioguanine nucleotides.

### Objectives

The primary objective of the 6MP study is to determine the objective tumour response rate to 6-mercaptopurine (6MP) and low-dose methotrexate in patients with previously treated breast, ovarian, fallopian tube or primary serous peritoneal cancer who are known to have a BRCA mutation, 8 weeks after starting treatment. Secondary objectives include progression-free survival (PFS), overall survival (OS), safety, toxicity, pharmacokinetics; and quality of life. Exploratory objectives include evaluation of patients’ Mismatch Repair (MMR) status at initial diagnosis, prior to starting the study, and after 7 weeks of treatment. An assessment of the feasibility of this trial in a multi-centre context will be used to inform the case for a follow-on phase III study.

## Methods

### Study design

The 6MP study is a single arm, non-randomised, two-stage, multi-centre Phase II trial. The trial opened in June 2011 and recruits from 14 centres across the UK. Following written informed consent and screening tests, eligible patients will commence treatment, taking 6MP once daily and methotrexate once weekly. Treatment will continue until disease progression or unacceptable toxicity. Conduct of the 6MP trial complies with the ethical principles of the Declaration of Helsinki (1996) and the regulatory requirements for clinical trials of an investigational medicinal product under the European Union Clinical Trials Directive. The study has been approved by the National Research Ethics Service (NRES) Committee South Central – Oxford B (REC reference 10/H0605/79).

The trial is sponsored by the University of Oxford, with funding from Cancer Research UK’s Clinical Trials Award and Advisory Committee (CTAAC) who approved a full funding application on 26th October 2010, and this work was supported by the Oxford Partnership Comprehensive Biomedical Research Centre with funding from the Department of Health’s National Institute of Health Research (NIHR) Biomedical Research Centre funding scheme.

Data submission for the 6MP trial is via electronic submission into the online database system OpenClinica by site staff, and this is closely monitored. The Oncology Clinical Trial Office (OCTO) coordinates the trial, and OCTO staff are in charge of the day-to-day running of the trial, as well as managing the trial as a whole; organising trial meetings, completing trial documentation, site monitoring, and database management. All statistical analysis will be undertaken by statisticians at the Centre for Statistics in Medicine (CSM) at the University of Oxford.

### Participants

To be eligible for inclusion in the 6MP study, patients must be aged 18 or older, have proven BRCA1 or BRCA2 mutations, and have measurable disease as defined by RECIST v1.1 criteria. For breast cancer patients, patients must have locally advanced or metastatic breast cancer, and may have received up to 3 previous lines of chemotherapy in the locally advanced or metastatic setting. Ovarian, fallopian tube or primary serous peritoneal cancer patients must have disease that is either platinum resistant or in whom further platinum based therapy is inappropriate. All patients must have an Eastern Cooperative Oncology Group (ECOG) performance score between 0 and 2, a life expectancy of greater than 12 weeks, and adequate haematological and biochemical function. Patients are excluded from participating in the study if they have a Low/Low genotype on Thiopurine methyltransferase (TPMT) testing. Patients with low activity (10% prevalence) or especially absent activity (prevalence 0.3%) are at a heightened risk of drug-induced bone marrow toxicity due to accumulation of the unmetabolised 6MP. At the start of the trial, patients were excluded from participation if they had low TPMT activity (<68 U/ml), but a decision was made by the 6MP TMG in October 2013 to remove this exclusion as a substantial amendment to the protocol, as evidence suggested that moderate TPMT activity levels did not affect dose intensity or levels of myelosuppression seen. Patients were already being closely monitored in the first cycle therefore no additional monitoring was implemented for this patient group. A total of 14 sites across the UK are actively recruiting for this trial; Royal United Hospital, Bath; Belfast City Hospital, Belfast; Velindre Hospital, Cardiff; University Hospital, Coventry and Warwickshire; Western General Hospital, Edinburgh; Guy’s Hospital, London; Royal Marsden Hospital, London; University College London Hospital, London; Christie Hospital, Manchester; Freeman Hospital, Newcastle; Mount Vernon Hospital, Middlesex; Churchill Hospital, Oxford; Royal Marsden Hospital, Sutton; Clatterbridge, Wirral.

### Interventions

Patients in the trial are treated with 6-mercaptopurine (6MP) and methotrexate continuously in 28 day cycles until disease progression or unacceptable toxicity. The dose of 6MP given is 55 mg/m^2^ body surface area, administered orally once a day at least one hour after eating; tablets should be taken at roughly the same time each day. A 15 mg/m^2^ dose of methotrexate should be taken orally once a week in the morning. It should be noted that these starting doses are not those originally set at the beginning of the trial. Doses were originally 75 mg/m^2^ of 6MP daily, and 20 mg/m^2^ of methotrexate weekly, but these were reduced following an Urgent Safety Measure in August 2012 due to a large proportion of patients requiring a dose reduction or treatment delay due to incidences of myelosuppression. Although myelosuppression is an expected toxicity of these agents and the Internal Safety Committee did not have serious safety concerns, the Trial Management Group concurred that a dose reduction was indicated to ensure dose intensity could be maintained without compromising patient safety.

### Outcomes and assessments

The primary outcome of the 6MP study is objective response, defined as complete response (CR), partial response (PR), or stable disease (SD), as measured by radiological disease response using RECIST criteria version 1.1. Patients who yield progressive disease (PD) are classed as non-responders. Tumour size is measured radiologically with computerised tomography (CT) and/or magnetic resonance imaging (MRI); the same method is used at baseline and at follow-up. The first time point for this primary analysis is 8 weeks after first treatment.

Secondary outcome measures include safety, overall survival (OS) and progression-free survival (PFS). Length of overall survival is defined in whole days as the time from entry into the study until death from any cause, and those patients who are not observed to die during the course of the trial will be censored at their last known follow-up date. Length of progression-free survival is defined in whole days as the time from entry into the study until progression or death from any cause, and those patients who are not observed to progress or die during the course of the trial will be censored at their last known progression- free follow-up date. Grade 3 or 4 Adverse Events (AEs), as defined by the National Cancer Institute (NCI) Common Terminology Criteria for Adverse Events (CTCAE) version 4.0, and abnormal clinical and laboratory findings will be collected at regular follow-up visits throughout the trial in order to evaluate safety. Pharmacokinetics are measured by the assessment of red blood cell 6-thioguanine nucleotide (6TGN) levels using a validated high-performance liquid chromatography (HPLC) assay, and the effect of pharmacogenomics on thiopurine metabolism is measured by assessing the TPMT genotype using a validated assay. Another secondary outcome is quality of life, which is evaluated using the EQ-5D questionnaire at baseline, 3 and 6 months, and at the end of treatment or 12 months, as well as using the ECOG performance status measure after each cycle.

Recruitment is monitored throughout the trial, in order to assess the feasibility of the 6MP study as a multi- centre study. If each site recruits 3–4 patients per year, this indicates that a large randomised phase III study would be feasible. The exploratory outcome measure of the 6MP trial is the Mismatch Repair (MMR) status of each patient; this will be assessed using validated immunohistochemical/MSI assays using tissue from initial diagnosis, just prior to starting the study, and after 7 weeks of treatment, as well as from optional extra biopsies.

### Sample size determination and power

The 6MP study aims to recruit 30 evaluable patients in the first stage, and 65 patients in total. The Simon compromise/admissible two-stage design [15, 16] was implemented to calculate this required sample size to detect an increase in response rate from 10% to 20%, with a power of 90% and a significance level of

20%. In the first stage of the study, if fewer than 3 out of 30 evaluable patients respond at 8 weeks after first treatment, then the trial will be stopped for futility; if 3 or more respond then a further 35 patients will be recruited in the second stage. If nine or more of the total 65 patients respond at 8 weeks then the treatment will be regarded as potentially effective and a phase III randomised trial will then be considered, if, in addition, the treatment appears to be safe and well tolerated, and the trial roll-out over multiple centres proves feasible.

### Statistical analysis

In the 6MP trial, fully evaluable patients (the per protocol population) are those patients who complete at least 4 out of 8 weeks of trial medication and are assessed for response as per RECIST v1.1 at 8 weeks. Patients who stop trial medication early due to disease progression (assessed by RECIST v1.1) are also fully evaluable because they have reached an end point. Partially evaluable patients (the intention to treat population) are those patients who have received at least one dose of treatment and who have a response assessment. Additional patients may be recruited at the discretion of the Chief Investigator to replace any that are not evaluable.

The proportion of patients responding to treatment will be presented, together with 95% confidence intervals, for Stage 1 (30 evaluable patients) and overall (65 evaluable patients). The number and proportion of patients who achieve CR, PR, SD and PD will also be reported, overall and separately for those patients who had received prior treatment with PARP inhibitors.

Overall survival and progression-free survival will both be presented overall and separately for patients previously treated with PARP inhibitors using Kaplan Meier graphs, and summarised as 1 and 2 year survival, together with their two-sided 95% confidence intervals. Median and quartile OS and PFS will also be presented. If the assumption of proportional hazards is met, the univariate hazard ratio (HR) from the Cox proportional hazards regression model will be given. In the case where the assumption of proportional hazards is inappropriate, parametric hazard functions will be considered.

Quality of life data will be presented descriptively overall and separately for patients previously treated with PARP inhibitors. Toxicity levels will be described using the NCI CTCAE version 4.0. Adverse Events (AEs) and Serious Adverse Events (SAEs) will be summarised by incidence rates and classified by the worst severity grade observed.

### Interim analysis

A safety interim analysis was undertaken 3 months after the first 12 patients were recruited into the trial and had received at least one dose of the trial drugs. No issues with safety were found in this review, and any observed side effects were found to be as expected for this patient group. Another interim analysis was carried out 8 weeks after the first 30 patients were recruited (at the end of stage one), and again no safety concerns were raised and the events reported were found to be as expected. Also at this interim analysis stage, the first milestone was reached and passed, and a further 35 patients are now currently being recruited.

### Monitoring committees

The Independent Early Phase Trials Oversight Committee (IEPTOC) are responsible for providing overall supervision of the trial; in particular trial progress, adherence to protocol, patient safety and consideration of new information. An Internal Safety Monitoring Committee is also in place for 6MP, consisting of the chief investigator, trial statistician, a member of the 6MP trial office, and an independent oncologist. This committee evaluate the ongoing safety of the trial participants, and also reviewed all safety interim analyses.

### Ethical approval

Obtained from the National Research Ethics Service (NRES) Committee South Central – Oxford B. Rec reference number 10/H0605/79.

## Discussion

The in vitro and in vivo findings of Issaeva and colleagues [14] suggest that 6TG might be an effective treatment in BRCA deficient tumours as a first line treatment or after developing resistance to PARP inhibitors or platinum drugs. 6TG is not recommended for continuous therapy due to its hepatotoxicity, but the antimetabolite 6MP, which is structurally similar to 6TG, has been shown to have comparable efficacy in patients with acute lymphoblastic leukaemia. To our knowledge, there have been no other studies comparing the efficacy of 6TG and 6MP in patients with solid malignancies. The aim of the 6MP trial is to evaluate the efficacy and safety of 6MP in combination with low dose methotrexate in patients with breast or ovarian cancer who are known to have a BRCA mutation, who have progressed after at least one previous line of chemotherapy. Exploiting the genetic basis of these tumours enables us to develop a more tailored approach to therapy for patients in this cohort.

## Confidentiality, dissemination of results and publication policy

Information collected during the trial is considered strictly confidential and will be securely stored with restricted access in accordance with the Data Protection Act. To protect the participant's identity, only initials and date of birth along with an assigned trial number will be used as patient identifiers. It is clearly stated in lay terms in the Patient Information Sheet and as a separate point within the Consent Form that authorised staff from the local Trust R&D office, the research team based at each study site, OCTO personnel during on site monitoring visits, and auditors from regulatory bodies may have access to their patient records.

The Sponsor recognises that participating Investigators have a responsibility under the Research Governance Framework for Health and Social Care to ensure that results of scientific interest arising from the Study are appropriately published and disseminated. The Chief Investigator will ensure public disclosure of the clinical trial research results in the form of a peer reviewed scientific journal publication and for other academic research purposes as appropriate. Participating site investigators will be provided with the full summary of results and encouraged to share the summary of results with study participants, as appropriate.

## Trial status

The trial is open for recruitment at the time of manuscript submission.

## Abbreviations

6MP: 6-mercaptopurine
6TGN: 6-thioguanine nucleotide
AE: Adverse Event
BER: Base excision pathway
CT: Computed tomography
CTCAE: Common Terminology Criteria for Adverse Events
ECOG: Eastern Cooperative Oncology Group
HPLC: High-performance liquid chromatography
FIGO: International Federation of Gynaecology and Obstetrics
IEPTOC: Independent Early Phase Trial Oversight Committee
MMR: Mismatch Repair
MRI: Magnetic resonance imaging
NCI: National Cancer Institute
OS: Overall Survival
PARP: Poly (ADP-ribose) polymerase
PFS: Progression-Free Survival
RECIST: Response evaluation criteria in solid tumours
SAE: Serious Adverse Event
TPMT: Thiopurine methyltransferase.
